# TIMP3 promotes the maintenance of neural stem-progenitor cells in the mouse subventricular zone

**DOI:** 10.3389/fnins.2023.1149603

**Published:** 2023-06-28

**Authors:** Lingyan Fang, Takaaki Kuniya, Yujin Harada, Osamu Yasuda, Nobuyo Maeda, Yutaka Suzuki, Daichi Kawaguchi, Yukiko Gotoh

**Affiliations:** ^1^Graduate School of Pharmaceutical Sciences, The University of Tokyo, Tokyo, Japan; ^2^Department of Sports and Life Sciences, National Institute of Fitness and Sports in Kanoya, Kanoya, Japan; ^3^Department of Computational Biology and Medical Sciences, Graduate School of Frontier Sciences, The University of Tokyo, Chiba, Japan; ^4^International Research Center for Neurointelligence (WPI-IRCN), The University of Tokyo, Tokyo, Japan

**Keywords:** TIMP3, adult neural stem cell, embryonic neural stem-progenitor cell, stem cell maintenance, notch signaling

## Abstract

Adult neural stem cells (NSCs) in the mouse subventricular zone (SVZ) serve as a lifelong reservoir for newborn olfactory bulb neurons. Recent studies have identified a slowly dividing subpopulation of embryonic neural stem-progenitor cells (NPCs) as the embryonic origin of adult NSCs. Yet, little is known about how these slowly dividing embryonic NPCs are maintained until adulthood while other NPCs are extinguished by the completion of brain development. The extracellular matrix (ECM) is an essential component of stem cell niches and thus a key determinant of stem cell fate. Here we investigated tissue inhibitors of metalloproteinases (TIMPs)—regulators of ECM remodeling—for their potential roles in the establishment of adult NSCs. We found that *Timp2*, *Timp3*, and *Timp4* were expressed at high levels in slowly dividing NPCs compared to rapidly dividing NPCs. Deletion of TIMP3 reduced the number of adult NSCs and neuroblasts in the lateral SVZ. In addition, overexpression of TIMP3 in the embryonic NPCs suppressed neuronal differentiation and upregulated the expression levels of Notch signaling relating genes. These results thus suggest that TIMP3 keeps the undifferentiated state of embryonic NPCs, leading to the establishment and maintenance of adult NSCs.

## 1. Introduction

In the subventricular zone (SVZ) of the adult mouse brain, neural stem cells (NSCs) remain in the quiescent state and produce neurons throughout life (Morshead et al., 1994; Doetsch and Alvarez-Buylla, 1996; Doetsch et al., 1999). Once activated, adult NSCs generate transit-amplifying progenitors (TAPs) and then differentiate into neuroblasts. Neuroblasts later migrate into the olfactory bulb and differentiate into interneurons, which integrate into existing circuitry and modify innate and cognitive functions (Bond et al., 2015; Lledo and Valley, 2016). The mechanism by which adult NSCs are established and maintained during development is under exploration. Recent studies have revealed that a slowly dividing subpopulation of neural stem-progenitor cells (NPCs) is set aside during development and later become adult NSCs in the SVZ, while other NPCs divide rapidly and contribute to brain development by generating neurons and glial cells (Fuentealba et al., 2015; Furutachi et al., 2015). Compared with the rapidly dividing NPCs that are extinguished after development, slowly dividing NPCs must be maintained for a longer period to generate adult NSCs. However, the regulatory mechanisms that preferentially maintain slowly dividing NPCs are not fully understood.

Stem cells are maintained in specialized microenvironments, or niches. In adult SVZ, niche-specific extracellular matrix (ECM) has been characterized (Kerever et al., 2007; Kjell et al., 2020). Given the crucial roles of the ECM in regulating NSC fate (Kazanis and Ffrench-Constant, 2011; Faissner and Reinhard, 2015; Long and Huttner, 2019), we hypothesized that factors which modify the ECM may be important for the long-term maintenance of slowly dividing NPCs. ECM remodeling is controlled by the balance between matrix metalloproteinases (MMPs) and tissue inhibitors of metalloproteinases (TIMPs). MMPs degrade ECM proteins and promote ECM turnover (Nagase and Woessner, 1999; Page-McCaw et al., 2007), while TIMPs function as endogenous inhibitors of MMPs (Brew and Nagase, 2010; Murphy, 2011). Increasing evidence has indicated the roles of MMPs in the regulation of postnatal NSC proliferation in the SVZ. MMP12 regulates postnatal ECM organization and NSC proliferation (Shan et al., 2018). MT5-MMP (MMP24) controls NSC quiescence by cleaving N-cadherin (Porlan et al., 2014). However, the function of TIMPs in embryonic NPCs or adult NSCs remains largely unknown.

Here we found that TIMP3 is expressed at a higher level in slowly dividing NPCs than in rapidly dividing NPCs. Deletion of TIMP3 reduced the number of adult NSCs in the lateral SVZ. Meanwhile, overexpression of TIMP3 suppressed neuronal differentiation of embryonic NPCs. We also found that TIMP3 overexpression enhanced the expression of genes related to Notch signaling pathway. Our findings thus uncover a physiological role for TIMP3 in the maintenance of neural stem-progenitor cells.

## 2. Materials and methods

### 2.1. Mice

Slc:ICR (ICR) and C57BL/6JJcl (B6J) mice were obtained from SLC Japan and CLEA Japan. *Rosa26-rtTA* (#006965) and *TRE-mCMV-H2B-GFP* mice (#005104) were obtained from The Jackson Laboratory. *Timp3* KO mice were produced using the gene-targeting technique described previously (Kawamoto et al., 2006). Briefly, mice carrying the mutant allele were backcrossed with B6J mice to generate KO mice in a B6J background. All mice were maintained in a temperature- and relative humidity-controlled (23 ± 3°C and 50 ± 15%, respectively) environment with a normal 12-h light/dark cycle. They were housed two to six per sterile cage (Innocage, Innovive) with chips (PALSOFT, Oriental Yeast), and with irradiated food (CE-2, CLEA Japan) and filtered water available *ad libitum*. Mouse embryos were isolated at various ages, with E0.5 being considered the time of vaginal plug appearance. All animals were maintained and studied according to protocols approved by the Animal Care and Use Committee of The University of Tokyo.

### 2.2. Plasmid constructs

EGFP sequence was inserted into pCAGEN to generate pCAGEN-EGFP. pCAG2-IRES-EGFP (pCAG2IG) was used as previously described (Kawai et al., 2017). The coding sequence of mouse *Timp3* was inserted into pCAGEN and pCAG2IG to generate pCAGEN-TIMP3 and pCAG2IG-TIMP3, respectively.

### 2.3. Injection of 9 TB-dox

9-*tert*-Butyl Doxycycline (9 TB-Dox) HCl (Echelon Biosciences) was dissolved in water to a final concentration of 2 μg/μL. 240–300 μg was injected intraperitoneally.

### 2.4. Flow cytometry

The lateral ganglionic eminences (LGEs) were dissected and subjected to enzymatic digestion with a papain-based solution (Wako). Dissociated cells were incubated first for 15 min on ice in 0.2% bovine serum albumin (BSA)/phosphate-buffered saline (PBS) with primary antibodies—PE/Cy7 anti-CD133 (1:100, BioLegend Cat# 141210, RRID:AB_2564069), APC anti-CD24 (1,100, BioLegend Cat# 101814, RRID:AB_439716), and anti-Isolectin B_4_ (biotin conjugate) (1,1,000, Sigma-Aldrich Cat# L2140, RRID:AB_2313663)—and then for 5 min on ice in 0.2% BSA/PBS with Streptavidin PE (1,500, eBioscience Cat# 12-4317-87). Cells were sorted on FACSAria IIIu (BD Bioscience). Debris and aggregated cells were removed by gating based on forward and side scatter.

### 2.5. Quantitative RT-PCR analysis

Total RNA was isolated from sorted NPCs using RNAiso Plus (Takara). Reverse transcription (RT) was performed with a maximum of 0.5 μg of total RNA using ReverTra Ace qPCR Master Mix with gDNA remover (TOYOBO). The resulting cDNA was subjected to real-time PCR analysis in LightCycler 480 II (Roche) with KAPA SYBR Fast qPCR Kit (NIPPON Genetics). The amount of target mRNA was normalized by that of *Actb* mRNA. Primer sequences were as follows:

*Actb*

Forward: 5’-AATAGTCATTCCAAGTATCCATGAAA-3’

Reverse: 5’-GCGACCATCCTCCTCTTAG-3’.

*Timp2*

Forward: 5’-GTTGGAGGAAAGAAGGAGTATCTAA-3’

Reverse: 5’-ACAATGAAGTCACAGAGGGTA-3’.

*Timp3*

Forward: 5’-CCTGGCTATCAGTCCAAAC-3’

Reverse: 5’-GTTGCTGATGCTCTTGTCT-3’.

*Timp4*

Forward: 5’-ATCCATCTGTGCAACTACATT-3’

Reverse: 5’-GTTCTGGTGGTAGTGATGATTC-3’.

*Sdc2*

Forward: 5’-CTCATGGTGTCTGTCAATCA-3’

Reverse: 5’-CCAAATACATGCAGAATAACAATACTT-3’.

### 2.6. Immunohistofluorescence analysis

Mice were subjected to perfusion fixation with ice-cold 4% paraformaldehyde (Merck) in PBS. The brain was isolated and exposed to the same fixative at 4°C for 120 min (postnatal) or 90 min (embryonic), equilibrated with 30% (w/v) sucrose in PBS, embedded in OCT compound (Tissue TEK), and frozen. Coronal sections (thickness of 12 μm) were exposed to Tris-buffered saline containing 0.1% Triton X-100 and 3% bovine serum albumin (blocking solution) for 2 h at room temperature, then incubated overnight at 4°C with primary antibodies in blocking solution and for 2 h at room temperature with Alexa Fluor-conjugated secondary antibodies (1:500, Thermo Fisher Scientific) and Hoechst 33342 (1:2000, Molecular Probes) in blocking solution, finally mounted in Mowiol (Calbiochem). Images were obtained with a laser confocal microscope (Leica TCS-SP5, Leica Mica, or Zeiss LSM 880) and were processed with LAS AF (Leica), ZEN (Zeiss), and Fiji (U.S. National Institutes of Health) software. Primary antibodies were as follows: anti-Ascl1 (1:500, BD Biosciences Cat# 556604, RRID:AB_396479), anti-Collagen IV (1:50, Abcam Cat# ab6586, RRID:AB_305584), anti-Dcx (1:1000, Abcam Cat# ab18723, RRID:AB_732011), anti-EGFR (1:500, Fitzgerald Industries International Cat# 20-ES04, RRID:AB_231428), anti-GFAP (1:1000, Abcam Cat# ab4674, RRID:AB_304558), anti-GFP (1:2000, Abcam Cat# ab13970, RRID:AB_300798; 1:1000, Nacalai Tesque Cat# GF090R, RRID:AB_2314545), anti-Ki67 (1:500, Agilent Cat# M7249, RRID:AB_2250503), anti-Sox2 (1:200, Cell Signaling Technology Cat# 3728, RRID:AB_2194037; 1:500, Santa Cruz Biotechnology Cat# sc-17320, RRID:AB_2286684), anti-S100β (1:200, Sigma-Aldrich Cat# S2657, RRID:AB_261477), anti-Tbr2 (1:1000, Millipore Cat# AB15894, RRID:AB_10615604; 1:500, Abcam Cat# ab23345, RRID:AB_778267), and anti-Tenascin C (1:50, Abcam Cat# ab108930, RRID:AB_10865908).

### 2.7. Administration of thymidine analogs

For identifying slowly dividing NPCs, 5-ethynyl-2’-deoxyuridine (EdU, Invitrogen, 5 mg/kg body weight) was injected intraperitoneally four times at 3-h intervals at embryonic day (E) 10.5. EdU was detected using Click-iT Plus EdU Cell Proliferation Kit for Imaging (Invitrogen).

### 2.8. *In utero* electroporation

The introduction of plasmid DNA into NPCs in the embryonic brain was performed as previously described (Tabata and Nakajima, 2001). In brief, plasmid DNA was injected into the lateral ventricle with an injector (FemtoJet, Eppendorf), electrodes were positioned at the flanking ventricular regions, and four–eight pulses of 35–45 V for 50 ms were applied at intervals of 950 ms using an electroporator (CUY21, NEPA GENE). The uterine horn was returned to the abdominal cavity so that the embryos continued to develop. The pCAGEN-EGFP plasmid was used to identify successfully electroporated cells.

### 2.9. RNA-sequencing (Quartz-Seq) analysis

RNA extraction, reverse transcription, and amplification of cDNA were performed on 1,000 cells aliquoted by FACS as described previously (Sasagawa et al., 2013). In brief, total RNA was purified using AMPure XP RNA cDNA (Beckman) and was subjected to reverse transcription with SuperScript III (Thermo Scientific). The cDNA was purified using AMPure XP (Beckman). Primers were digested by adding ExoI (Takara). Poly-A tail was added with terminal deoxynucleotidyl transferase (Roche). The cDNA was amplified using MightyAmp DNA polymerase (Takara) and was purified using PCR Extraction Kit (Nippon Genetics). Sequence data were obtained with a 36-base single-end on the Illumina HiSeq2500 platform. Approximately 2–4 million sequences were obtained from each sample. Sequences were mapped to the mouse genome (mm9) using ELAND v2 (Illumina). Only uniquely mapped reads with no base mismatches were used. Reads were normalized by TMM (weighted trimmed mean of M-values) normalization (Robinson and Oshlack, 2010) as implemented by the R package *edgeR* (RRID:SCR_012802) (Robinson et al., 2009). Differential gene expression analysis was performed using *edgeR*. Reads per kilobase of mRNA model per million total reads (RPKM) was calculated to analyze gene expression levels. Processed data of RNA sequence was shown in Supplementary Table 1. Gene ontology and pathway enrichment analysis were conducted with WebGestalt: WEB-based GEne SeT AnaLysis Toolkit (RRID:SCR_006786) (Liao et al., 2019).

### 2.10. Statistical analysis

Quantitative data are presented as means ± SEM and were compared with the two-tailed paired *t* test or the two-tailed Student’s *t* test as indicated using GraphPad Prism (RRID:SCR_002798). A *p* value of <0.05 was considered statistically significant. The number of animals in each experiment is stated in the respective figure legends.

## 3. Results

### 3.1. *Timp2, Timp3*, and *Timp4* are highly expressed in slowly dividing embryonic NPCs and adult quiescent NSCs

**Figure 1:**
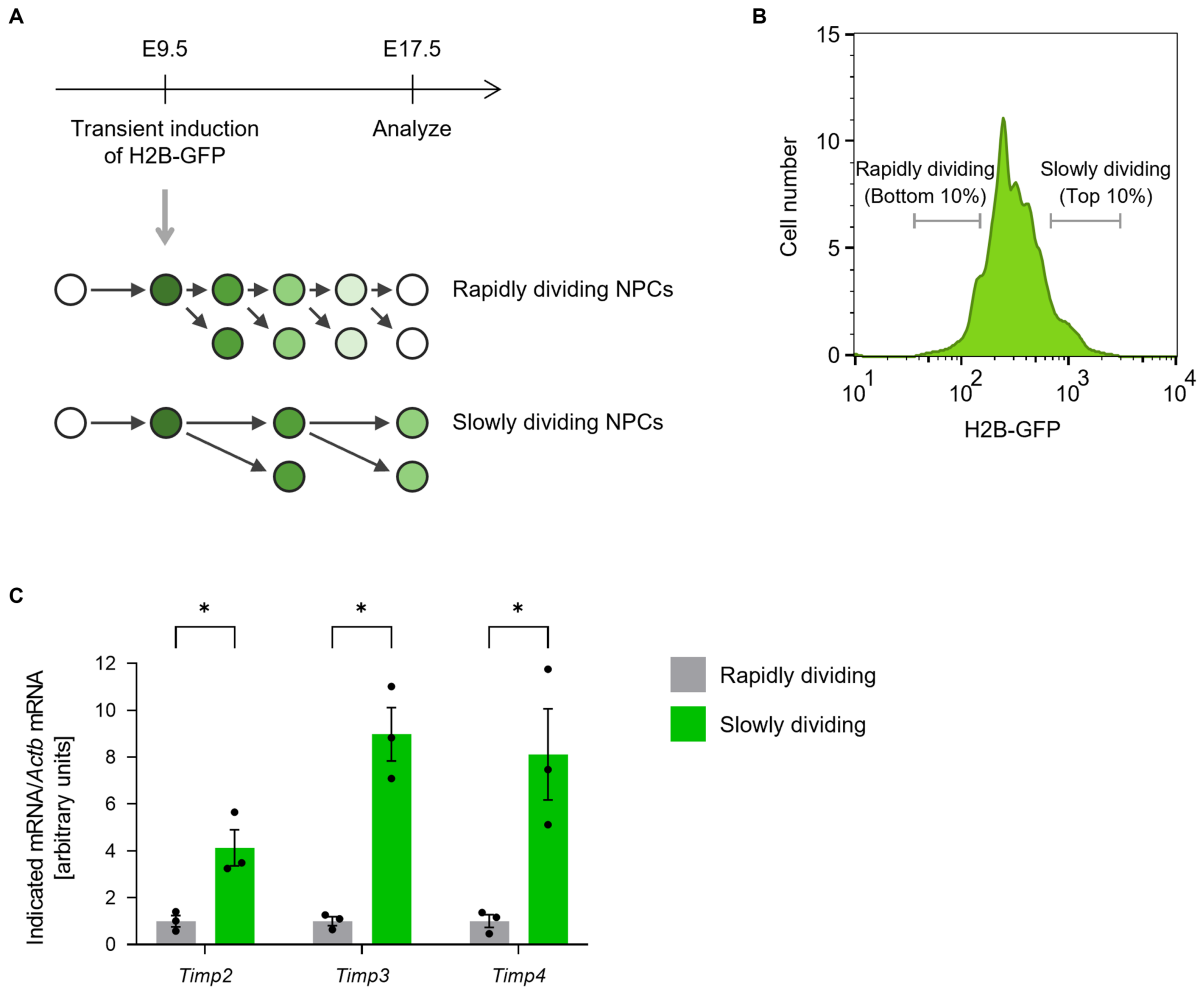
*Timp2*, *Timp3*, and *Timp4* are highly expressed in slowly dividing NPCs. **(A)** The scheme for isolating rapidly dividing and slowly dividing NPCs. H2B-GFP was transiently induced at E9.5 and the LGE was dissected at E17.5. NPCs were defined as cells positive for NPC marker CD133, negative for neuronal marker CD24, and negative for endothelial marker Isolectin B_4_. **(B)** Representative flow cytometric histogram of H2B-GFP fluorescence intensity. Rapidly dividing NPCs (bottom 10% of NPCs for H2B-GFP intensity) and slowly dividing NPCs (top 10% of NPCs for H2B-GFP intensity) were collected. **(C)** Quantitative RT-PCR analysis of *Timp2*, *Timp3*, and *Timp4* mRNAs. Data are means ± SEM (*n* = 3 independent experiments). *p < 0.05 by two-tailed paired *t* test.

We first compared the expression levels of TIMP family members between rapidly and slowly dividing NPCs in the lateral ganglionic eminences (LGEs). To monitor cell division frequency, we performed a histone 2B (H2B)-GFP retention analysis (Furutachi et al., 2015). Ubiquitous H2B-GFP expression was transiently induced at E9.5 by a single 9-tert-butyldoxycycline (9 TB-Dox) injection into pregnant *Rosa-rtTA;TRE-mCMV-H2B-GFP* mice. At E17.5, we collected two populations of CD133^+^CD24^−^Isolectin B_4_^−^ NPCs from the LGE according to H2B-GFP fluorescence intensity: the bottom 10% as the rapidly dividing NPCs and the top 10% as the slowly dividing NPCs (Figures 1A,B). Quantitative RT-PCR analysis revealed that the abundance of *Timp2*, *Timp3*, and *Timp4* mRNAs was significantly higher in slowly dividing NPCs than in rapidly dividing NPCs (Figure 1C).

**Figure 2:**
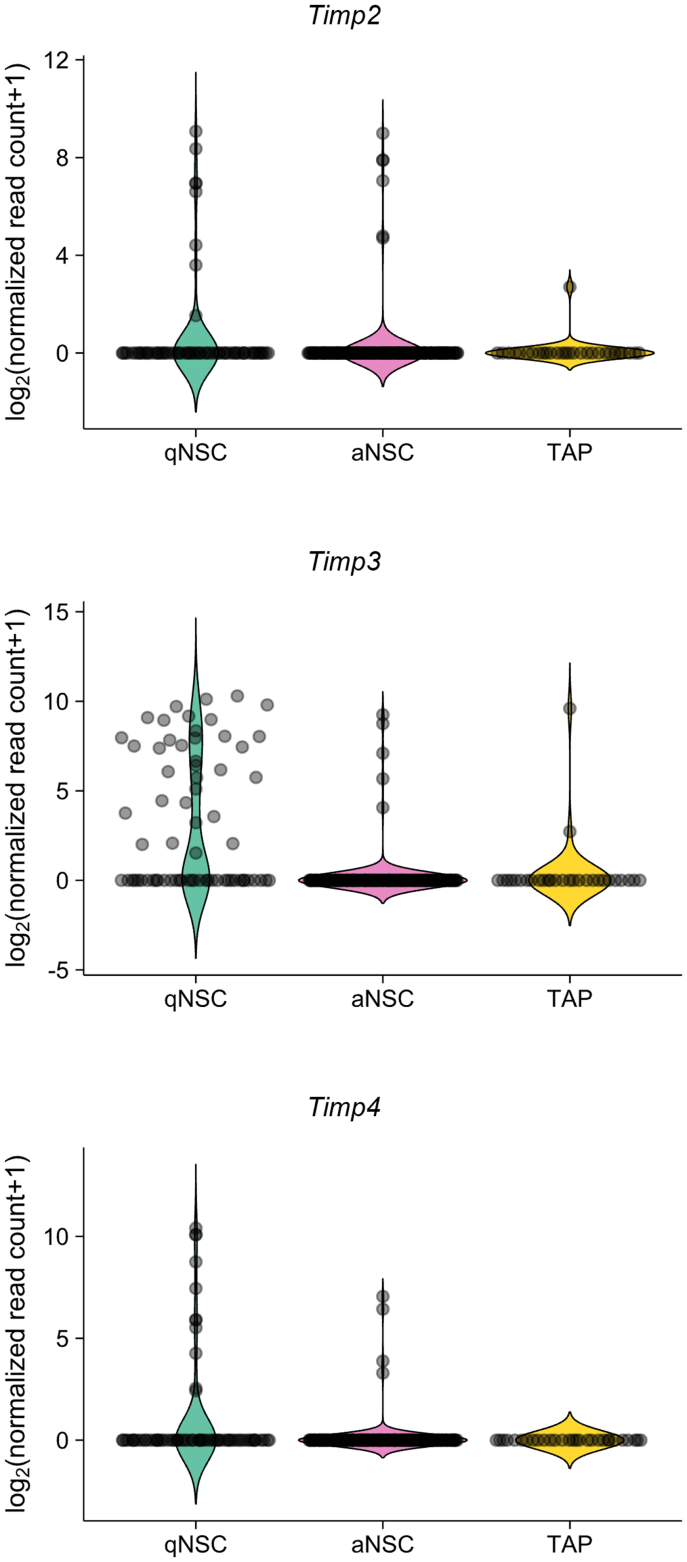
*Timp2*, *Timp3*, and *Timp4* are highly expressed in qNSCs in the adult SVZ. Expression of *Timp2*, *Timp3*, and *Timp4* in qNSCs, aNSCs, and TAPs (NPCs) from the SVZs of adult mice. Data were obtained from the previous study (Dulken et al., 2017).

We also analyzed the expression levels of TIMPs in the adult SVZ. A previous study has performed single-cell RNA-sequencing from quiescent NSCs (qNSCs), activated NSCs (aNSCs), and TAPs from SVZs of young adult (3 months old) mice (Dulken et al., 2017). Reanalyzing the data, we found that *Timp2*, *Timp3*, and *Timp4* were expressed relatively at high levels in qNSCs compared to aNSCs and TAPs (Figure 2). Together, these results suggest that *Timp2*, *Timp3*, and *Timp4* are highly expressed in quiescent subpopulations of both embryonic NPCs and adult NSCs.

### 3.2. TIMP3 contributes to the establishment or maintenance of adult NSCs in the lateral SVZ

**Figure 3:**
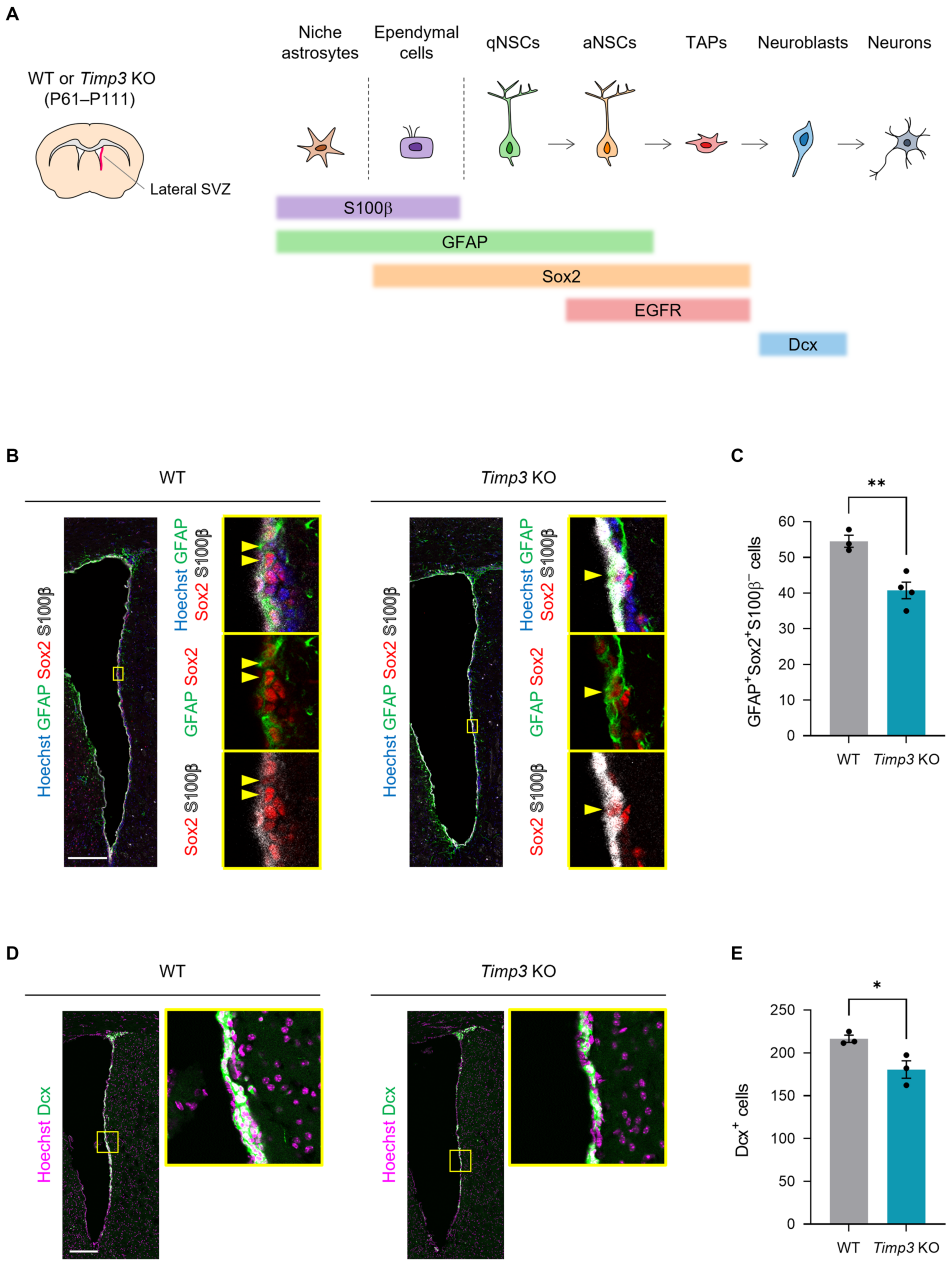
TIMP3 deletion reduces adult NSCs and neuroblasts. **(A)** Schematic showing the location of lateral SVZ and the makers for each cell type. WT mice and *Timp3* KO mice were sacrificed at P61–P111. The lateral wall of the SVZ was analyzed. **(B)** Immunohistofluorescence analysis of GFAP, Sox2, and S100β. Nuclei were stained with Hoechst 33342. Scale bar: 200 μm. Arrowheads indicate GFAP^+^Sox2^+^S100β^−^ adult NSCs. **(C)** Quantification of GFAP^+^Sox2^+^S100β^−^ adult NSCs. Data are means ± SEM (*n* = 3 and 4 animals for WT and *Timp3* KO, respectively). **p < 0.01 by two-tailed Student’s *t* test. **(D)** Immunohistofluorescence analysis of Dcx. Nuclei were stained with Hoechst 33342. Scale bar: 200 μm. **(E)** Quantification of Dcx^+^ neuroblasts. Data are means ± SEM (*n* = 3 and 3 animals for WT and *Timp3* KO, respectively). *p < 0.05 by two-tailed Student’s *t* test.

We next sought to examine the role of TIMPs in the genesis of adult NSCs. Here, we focused on TIMP3, given that TIMP2 has been shown to promote neuronal differentiation (Pérez-Martínez and Jaworski, 2005) and that TIMP4 is expressed relatively at a low level in NPCs in the embryonic mouse forebrain compared to TIMP2 and TIMP3 (La Manno et al., 2021). Since slowly dividing embryonic NPCs give rise to the majority of adult NSCs in the lateral wall of the SVZ (Furutachi et al., 2015), We examined whether TIMP3 is required for generating adult NSCs in the corresponding area (Figure 3A). We used *Timp3* knockout (KO) mice (postnatal day (P) 61–P111), which are viable and fertile and develop with no overt abnormalities (Kawamoto et al., 2006). Crucially, *Timp3* KO mice showed a reduced number of GFAP^+^Sox2^+^S100β^−^ adult NSCs in the lateral SVZ compared with wild-type (WT) mice (Figures 3B,C). We further followed the effects of TIMP3 deletion on descendants of NSCs. Although the number of GFAP^−^EGFR^+^S100β^−^ TAPs was not significantly changed (Supplementary Figures 1A,B), the number of Dcx^+^ neuroblasts was decreased in *Timp3* KO mice (Figures 3D,E). Therefore, TIMP3 appears to play an important role in increasing adult NSCs and neurogenesis in the lateral SVZ.

### 3.3. TIMP3 is not essential for the emergence of slowly dividing embryonic NPCs

**Figure 4:**
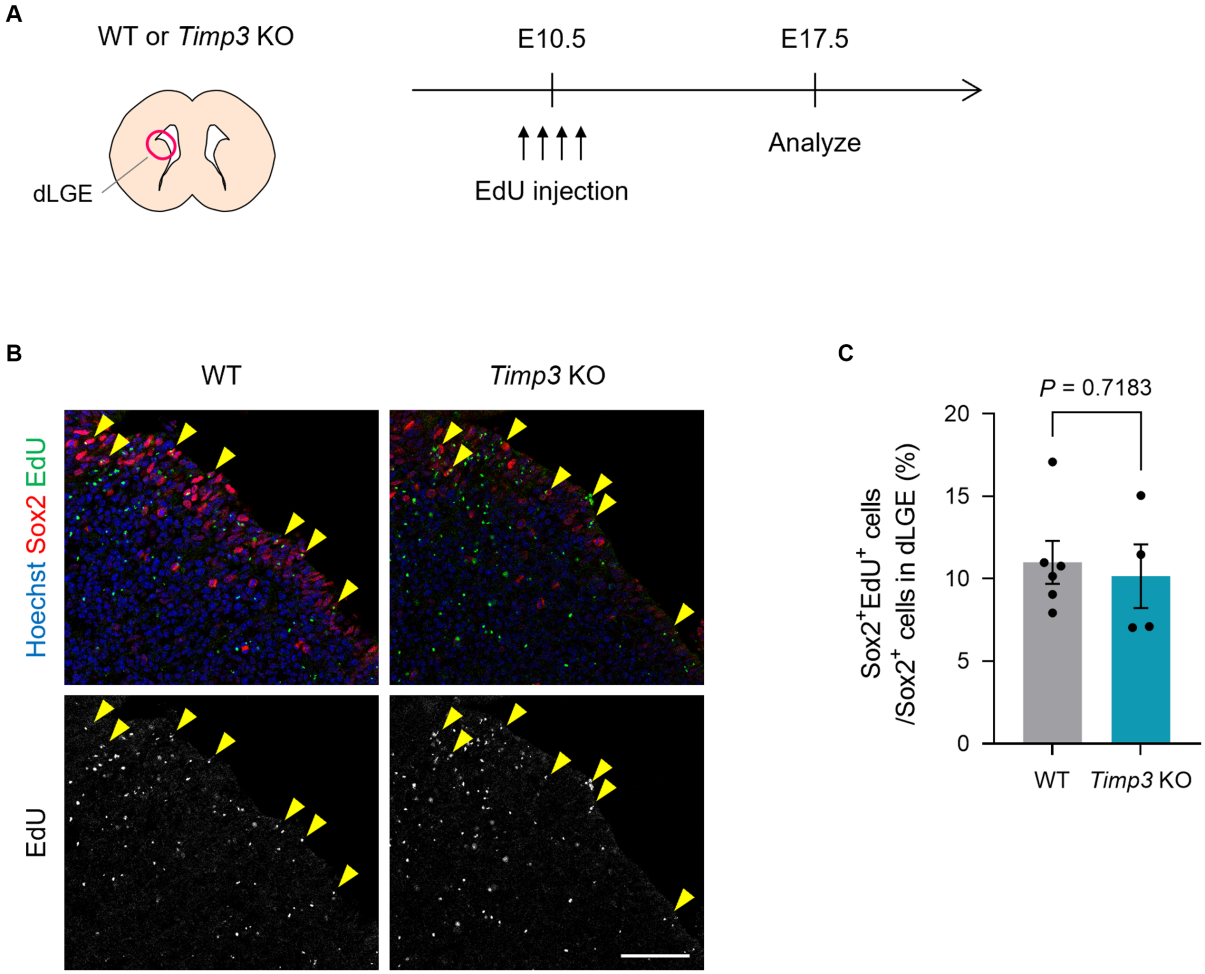
TIMP3 deletion does not change the number of slowly dividing embryonic NPCs. **(A)** Experimental design. EdU was injected intraperitoneally at E10.5 into pregnant WT and *Timp3* KO mice. Brain sections were obtained at E17.5 and dLGE was analyzed. **(B)** Immunohistofluorescence analysis of Sox2 and staining for EdU. Nuclei were stained with Hoechst 33342. Scale bar: 50 μm. Arrowheads indicate Sox2^+^EdU^+^ slowly dividing NPCs. **(C)** Quantification of EdU^+^ slowly dividing cells among Sox2^+^ NPCs in the dLGE. Data are means ± SEM (*n* = 6 and 4 embryos for WT and *Timp3* KO, respectively). Two-tailed Student’s *t* test.

We then asked whether TIMP3 regulates the emergence of slowly dividing embryonic NPCs. Using *Timp3* KO mice, we tested whether TIMP3 deletion affects the abundance of slowly dividing NPCs. We detected slowly dividing NPCs based on 5-ethynyl-2′-deoxyuridine (EdU) retention at E17.5 after injecting it into pregnant mice at E10.5 (Figure 4A). No significant difference, however, was found in the number of EdU-retaining slowly dividing Sox2^+^ NPCs in the dorsal LGE (dLGE) between WT mice and *Timp3* KO mice (Figures 4B,C). This result suggests that TIMP3 may not be required for the establishment of slowly dividing embryonic NPCs.

### 3.4. TIMP3 contributes to the maintenance of embryonic NPCs in the LGE

**Figure 5:**
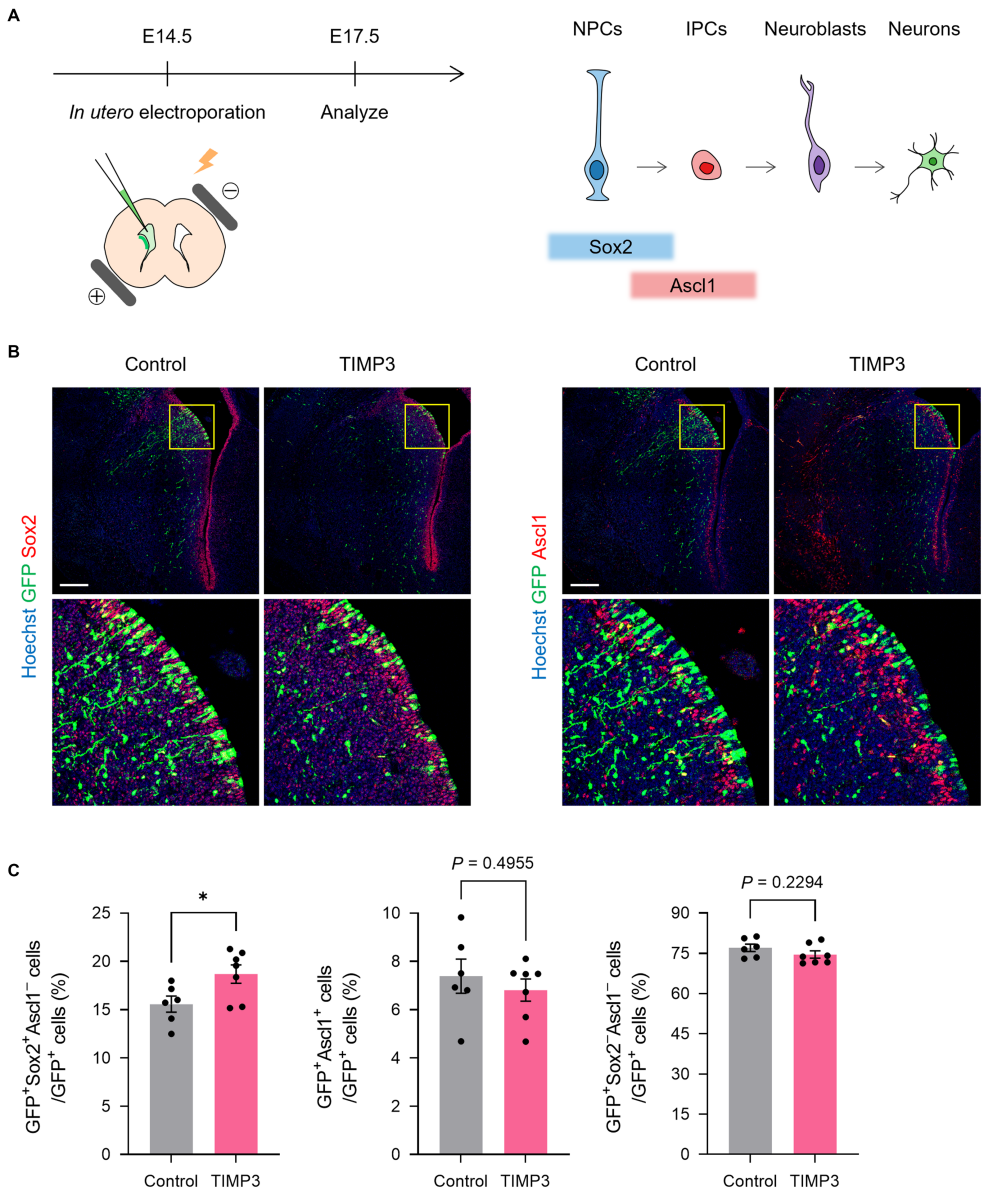
TIMP3 overexpression suppresses neuronal differentiation in the developing LGE. **(A)** Schematic showing the experimental design and the markers for each cell type. *In utero* electroporation was performed at E14.5 with plasmids expressing GFP, alone (control) or together with TIMP3. Embryos were analyzed at E17.5. NPC, neural progenitor cell; IPC, intermediate progenitor cell. **(B)** Immunohistofluorescence analysis of GFP, Sox2, and Ascl1. Nuclei were stained with Hoechst 33342. Scale bars: 200 μm. **(C)** Quantification of the proportion of Sox2^+^Ascl1^−^ cells, Ascl1^+^ cells, and Sox2^−^Ascl1^−^ cells among GFP^+^ cells. Data are means ± SEM (*n* = 6 and 7 embryos for control and *Timp3*, respectively). *p < 0.05 by two-tailed Student’s *t* test.

We then investigated the role for TIMP3 in the maintenance of slowly dividing NPCs. Slowly dividing embryonic NPCs are expected to be maintained in the undifferentiated state for a long period to become adult NSCs. We thus assumed that expression of TIMP3 at a high level might help maintain the undifferentiated state of slowly dividing NPCs. To test this possibility, we overexpressed TIMP3 in embryonic NPCs in the LGE at E14.5 by *in utero* electroporation (Figure 5A). Overexpression of TIMP3 significantly increased the percentage of Sox2^+^Ascl1^−^ undifferentiated cells at E17.5 (Figures 5B,C). This indicates the role of TIMP3 in maintaining the undifferentiated state of NPCs in the LGE.

### 3.5. TIMP3 promotes the maintenance of embryonic NPCs in the neocortex

**Figure 6:**
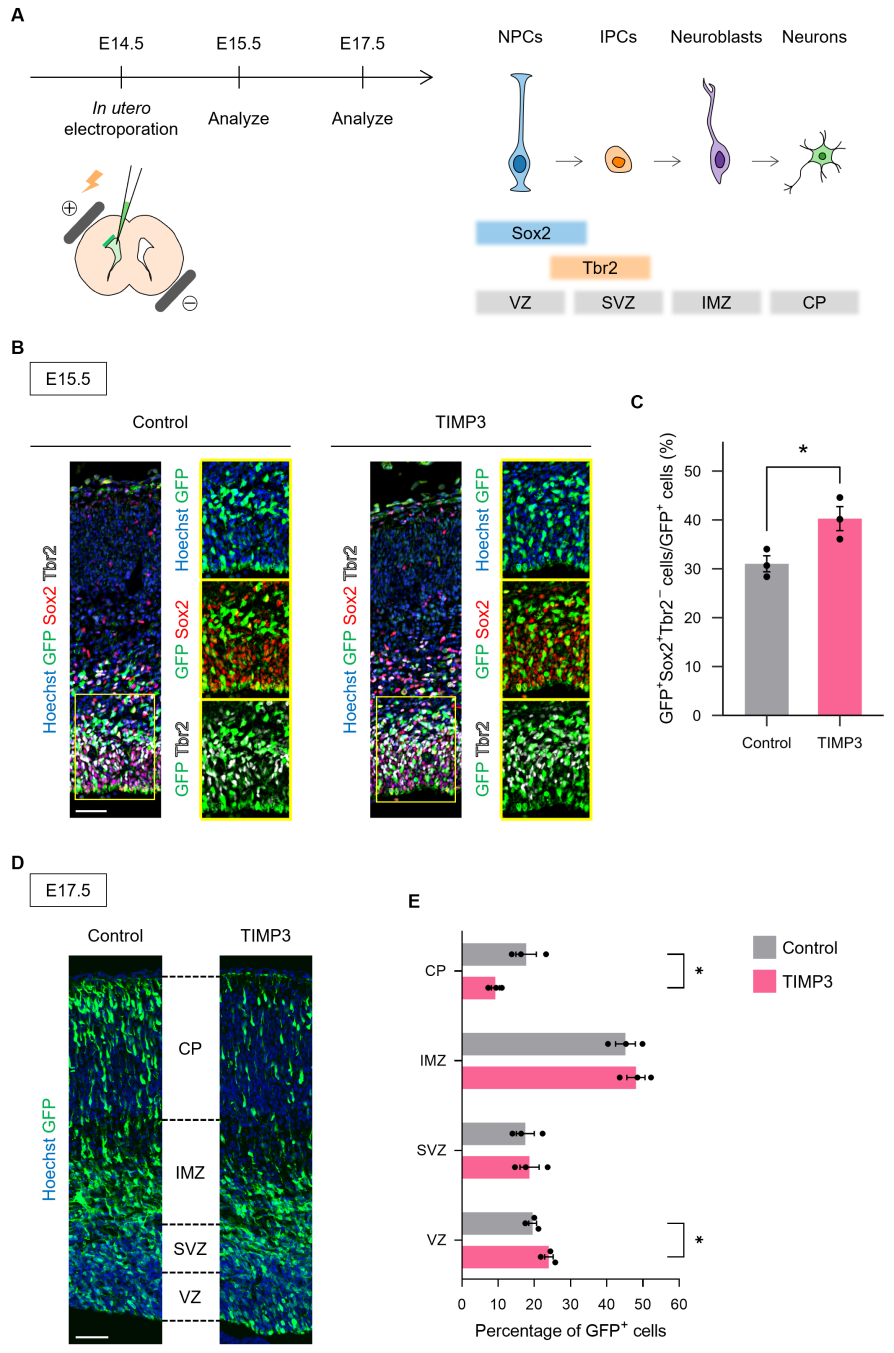
TIMP3 overexpression suppresses neuronal differentiation in the developing neocortex. **(A)** Schematic showing the experimental design and the markers for each cell type. *In utero* electroporation was performed at E14.5 with plasmids expressing GFP, alone (control) or together with TIMP3. NPC, neural progenitor cell; IPC, intermediate progenitor cell; VZ, ventricular zone; SVZ, subventricular zone; IMZ, intermediate zone; CP, cortical plate. **(B)** Embryos were subjected to immunohistofluorescence analysis of GFP, Sox2, and Tbr2 at E15.5. Nuclei were stained with Hoechst 33342. Scale bar: 50 μm. **(C)** Quantification of the proportion of Sox2^+^Tbr2^−^ cells in the VZ among GFP^+^ cells. Data are means ± SEM (*n* = 3 and 3 embryos for control and TIMP3, respectively). *p < 0.05 by two-tailed Student’s *t* test. **(D)** Embryos were subjected to immunohistofluorescence analysis of GFP at E17.5. Nuclei were stained with Hoechst 33342. Scale bar: 50 μm. **(E)** Distribution of GFP^+^ cells. Data are means ± SEM (*n* = 3 and 3 embryos for control and TIMP3, respectively). *p < 0.05 by two-tailed Student’s *t* test.

To test whether the maintenance of undifferentiated state mediated by TIMP3 can also be seen in other brain regions, we ectopically overexpressed TIMP3 in neocortical NPCs, in which the level of endogenous *Timp3* appears to be lower than that in NPCs located in the LGE (Figure 6A Supplementary Figure 2). Overexpression of TIMP3 at E14.5 increased the fraction of Sox2^+^Tbr2^−^ undifferentiated cells in the ventricular zone (VZ) (Figures 6B,C). Furthermore, we found that TIMP3 overexpression resulted in an increased proportion of GFP^+^ cells residing in the VZ and a reduced proportion of GFP^+^ cells in the cortical plate (CP) at E17.5 (Figures 6D,E). These results support the notion that TIMP3 promotes maintenance of the undifferentiated state of NPCs. Since cell cycle inhibition has been shown to promote NPC maintenance (Furutachi et al., 2015), we looked into the possible role of TIMP3 in regulating the cell cycle of NPCs. TIMP3 overexpression, however, did not change the fraction of cells positive for the proliferation marker Ki67 among Tbr2^−^ cells in the VZ (Supplementary Figures 3A,B). Thus, the increased proportion of cells remaining in the VZ was not likely attributable to cell cycle arrest.

**Figure 7:**
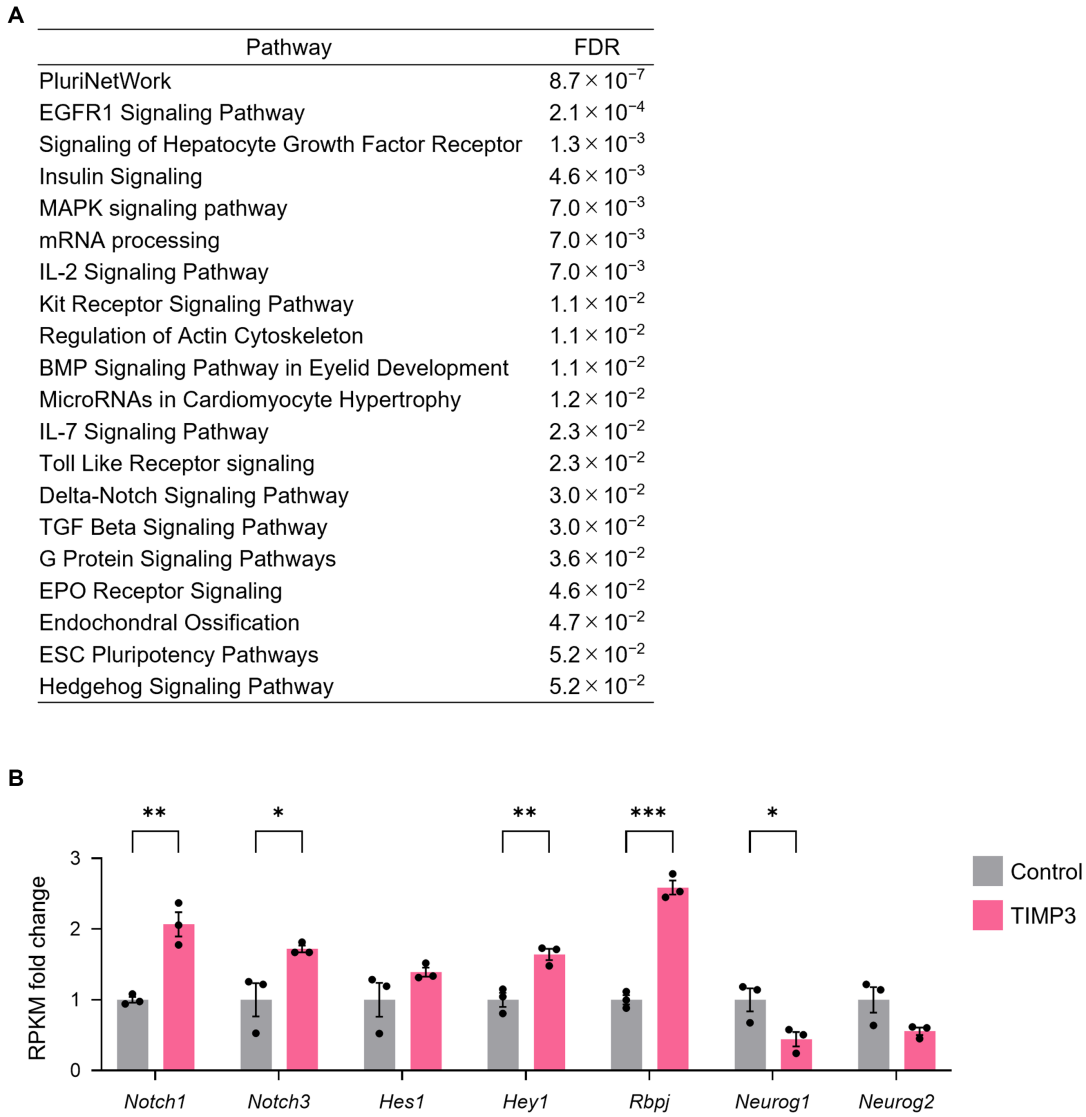
TIMP3 overexpression upregulates Notch signaling-related genes. **(A)** Pathway (WikiPathway) enrichment analysis of TIMP3-expressing NPCs enriched genes (*edgeR*; *p* < 0.05). The top 20 are ranked by FDR. **(B)** RPKM fold change relative to the average of control samples. Data are means ± SEM (*n* = 3 independent experiments). *p < 0.05, **p < 0.01, ***p < 0.001 by two-tailed Student’s *t* test.

To investigate the mechanism by which TIMP3 promotes NPC maintenance, we collected CD133^+^CD24^−^ NPCs positive for GFP by FACS from the neocortex of E17.5 embryos and performed RNA-sequencing (Quartz-Seq). 2,725 differentially expressed genes (DEGs) were found using the R package *edgeR* (Robinson et al., 2009), with 2,106 genes upregulated and 619 genes downregulated by TIMP3 overexpression (Supplementary Table 1). Pathway enrichment analysis of DEGs showed that genes upregulated in TIMP3-overexpreesed NPCs are enriched with those related to Delta-Notch signaling (Figure 7A). TIMP3 overexpression increased the levels of Notch receptors (*Notch1* and *Notch3*), Notch downstream effector (*Hey1*), and Notch pathway co-factor (*Rbpj*) (Figure 7B). Furthermore, overexpression of TIMP3 reduced the level of *Neurog1*, a proneural transcription factor negatively regulated by Notch signaling (Figure 7B). These results suggest that Notch signaling may be activated in TIMP3-overexpressed NPCs and keep their undifferentiated state.

## 4. Discussion

The developmental process leading to the establishment of adult tissue stem cells is a fundamental question. A slowly dividing subpopulation of NPCs has been identified as the embryonic origin of adult NSCs. The regulatory mechanism of this population, however, is not well understood. In the present study, we show that TIMP3 is preferentially expressed in slowly dividing NPCs—an embryonic origin of adult NSCs—compared with rapidly dividing NPCs—a cellular source of brain development. Deletion of TIMP3 reduced the adult NSC pool in the lateral SVZ, without changing the initial population of slowly dividing embryonic NPCs. These results implicate TIMP3 in the long-term maintenance of slowly dividing NPCs, an essential basis for the genesis of adult NSCs.

How then does TIMP3 mediate the long-term maintenance of neural stem-progenitor cells at a molecular level? Overexpression of TIMP3 in the embryonic NPCs suppressed neural differentiation both in the GE and in the neocortex. Importantly, TIMP3-overexpressed NPCs showed increased levels of *Notch1*, *Notch3*, and *Hey1*, suggesting that Notch signaling is activated in TIMP3-overexpressed NPCs. Notch signaling plays a crucial role in the maintenance of the undifferentiated state of both embryonic NPCs and adult NSCs (Gaiano et al., 2000; Imayoshi et al., 2010; Kawaguchi et al., 2013; Engler et al., 2018; Sueda et al., 2019; Zhang et al., 2019). High levels of active Notch1 and its downstream effector Hey1 in slowly dividing NPCs have been implicated in robust maintenance of the undifferentiated state from the embryonic to postnatal stages (Harada et al., 2021). Notch3 has been shown to be responsible for the maintenance of quiescent adult NSCs (Kawai et al., 2017; Than-trong et al., 2018). Our results thus suggest that TIMP3 may maintain the undifferentiated state of neural stem-progenitor cells potentially through the activation of Notch signaling.

Considering the canonical functions of TIMP3 in ECM remodeling via MMP inhibition, it is of interest that gene ontology and pathway enrichment analysis showed that upregulated genes in neocortical NPCs by TIMP3 overexpression include those related to Hippo signaling, indicating the possibility that ECM dynamics were altered by TIMP3 overexpression (Supplementary Figure 4). We performed immunostaining for Collagen IV and Tenascin C, two major ECM proteins expressed in embryonic NPCs. However, we could not detect obvious changes in their expression levels and distribution by TIMP3 overexpression (Supplementary Figures 5A,B). Thus, there might be other extracellular matrix proteins that are regulated by TIMP3. For example, *Sdc2* (coding for the ECM protein Syndecan2) is highly expressed in slowly dividing NPCs than in rapidly dividing NPCs (Supplementary Figure 6). We also found that the level of *Sdc2* mRNA was increased by TIMP3 overexpression (Supplementary Figure 7). Of note, Syndecan2 and Syndecan3 have been found to promote Notch signaling through direct interactions with Notch3 and Notch1, respectively (Pisconti et al., 2010; Zhao et al., 2012). It is thus possible that TIMP3 promotes the direct interactions between syndecans and Notch receptors and thereby activates Notch signaling (Supplementary Figure 8). Beside ECM remodeling, other mechanisms might link TIMP3 to the activation of Notch signaling. MT1-MMP (MMP14), a candidate target of TIMP3, is expressed in the mouse developing forebrain (La Manno et al., 2021) and has been reported to negatively regulate Notch signaling by cleaving the Notch ligand Dll1 (Jin et al., 2011; Jiang et al., 2020). Therefore, TIMP3 may activate Notch signaling *via* suppression of MT1-MMP. Future investigation of MMPs and ECM remodeling would provide more insight into downstream mechanisms.

The mechanisms responsible for different expression levels of TIMP3 between slowly and rapidly dividing NPCs remain unclear. Of note, previous RNA sequencing data (Harada et al., 2021) revealed that cyclin-dependent kinase inhibitor p57 overexpression increased the mRNA level of *Timp3* in neocortical NPCs (Supplementary Figure 9). Given that p57 is highly expressed in slowly dividing NPCs compared to rapidly dividing ones, it is possible that p57 itself or cell cycle arrest upregulates *Timp3* expression level in slowly dividing NPCs.

In conclusion, we have here unveiled the role of TIMP3 in the maintenance of embryonic NPCs and adult NSCs in the mouse subventricular zone. In the hippocampus, the other known neurogenic region of the adult mammal brain, *Timp3* was also enriched in quiescent NSCs and downregulated upon activation (Shin et al., 2015). Another study has reported that *Timp3* KO mice showed enhanced MMP activity in the hippocampus and delayed acquisition of spatial memory compared with WT mice (Baba et al., 2009). Since hippocampal neurogenesis is considered to be involved in memory formation, it will be of interest to examine the possible role of TIMP3 in hippocampal NSCs. Studies have shown the roles of TIMP3 in the regulation of other tissue stem cells as well. In mouse muscles, TIMP3 has been shown to suppress myogenic differentiation (Liu et al., 2010). The only *Drosophila timp* is required for normal oogenesis of the female germline stem cells (Pearson et al., 2016). Our findings thus provide novel evidence supporting the role of TIMP3 in long-term stem cell maintenance that may be shared by various adult tissue stem cells.

## Data availability statement

The authors acknowledge that the data presented in this study must be deposited and made publicly available in an acceptable repository, prior to publication. Frontiers cannot accept a manuscript that does not adhere to our open data policies. The sequence data have been deposited in the DNA Data Bank of Japan (DDBJ) Sequence Read Archive under the following accession number: DRA016558.

## Ethics Statement

The animal study was reviewed and approved by the Animal Care and Use Committee of the University of Tokyo.

## Author contributions

LF: conception and design, collection and assembly of data, data analysis and interpretation, and manuscript writing. TK: conception and design, collection and assembly of data, data interpretation, and supervision. YH: data interpretation and supervision. OY and NM: generating *Timp3* KO mice. YS: performing RNA sequencing experiments and analysis of RNA sequencing data. DK: data interpretation, financial support, and supervision. YG: conception and design, data interpretation, financial support, administrative support, supervision, and manuscript writing. All authors contributed to the article and approved the submitted version.

## Funding

This study was supported by the World-leading INnovative Graduate Study Program for Life Science and Technology of the University of Tokyo; by KAKENHI grants from the Ministry of Education, Culture, Sports, Science, and Technology of Japan and the Japan Society for the Promotion of Science (JP20J23093 to LF, JP16J03852 to TK, JP21K15180 to YH, JP21K06387 to DK, and JP15H05773, JP16H06279, JP16H06479, JP16H06481, JP22H00431, JP16H06279 to YG); by AMED-CREST of the Japan Agency for Medical Research and Development (JP19gm0610013, JP22gm1310004); by the Uehara Memorial Foundation; by the Japan Foundation for Applied Enzymology; and by the International Research Center for Neurointelligence (WPI-IRCN), the University of Tokyo Institutes for Advanced Study.

## Conflict of interest

The authors declare that the research was conducted in the absence of any commercial or financial relationships that could be construed as a potential conflict of interest.

## Publisher’s note

All claims expressed in this article are solely those of the authors and do not necessarily represent those of their affiliated organizations, or those of the publisher, the editors and the reviewers. Any product that may be evaluated in this article, or claim that may be made by its manufacturer, is not guaranteed or endorsed by the publisher.
