# Supplementary material for: TIMP3 promotes the maintenance of neural stem-progenitor cells in the mouse subventricular zone
Source: Front Neurosci. 2023 Jun 28;17:1149603. doi: 10.3389/fnins.2023.1149603 (PMC10338847; doi:10.3389/fnins.2023.1149603)
Supplement: Supplementary file 1 [file Data_Sheet_1.pdf]

Supplementary Material

TIMP3 promotes the maintenance of neural stem-progenitor cells in the mouse subventricular zone

Lingyan Fang, Takaaki Kuniya*, Yujin Harada, Osamu Yasuda, Nobuyo Maeda, Yutaka Suzuki, Daichi Kawaguchi, Yukiko Gotoh*

*** Correspondence:**
Takaaki Kuniya: taka1129@mol.f.u-tokyo.ac.jp
Yukiko Gotoh: ygotoh@mol.f.u-tokyo.ac.jp

1 Supplementary Data

Supplementary Table 1. Complete list of DEGs, related to Figure 7.

2 Supplementary Figures and Tables

2.1 Supplementary Figures

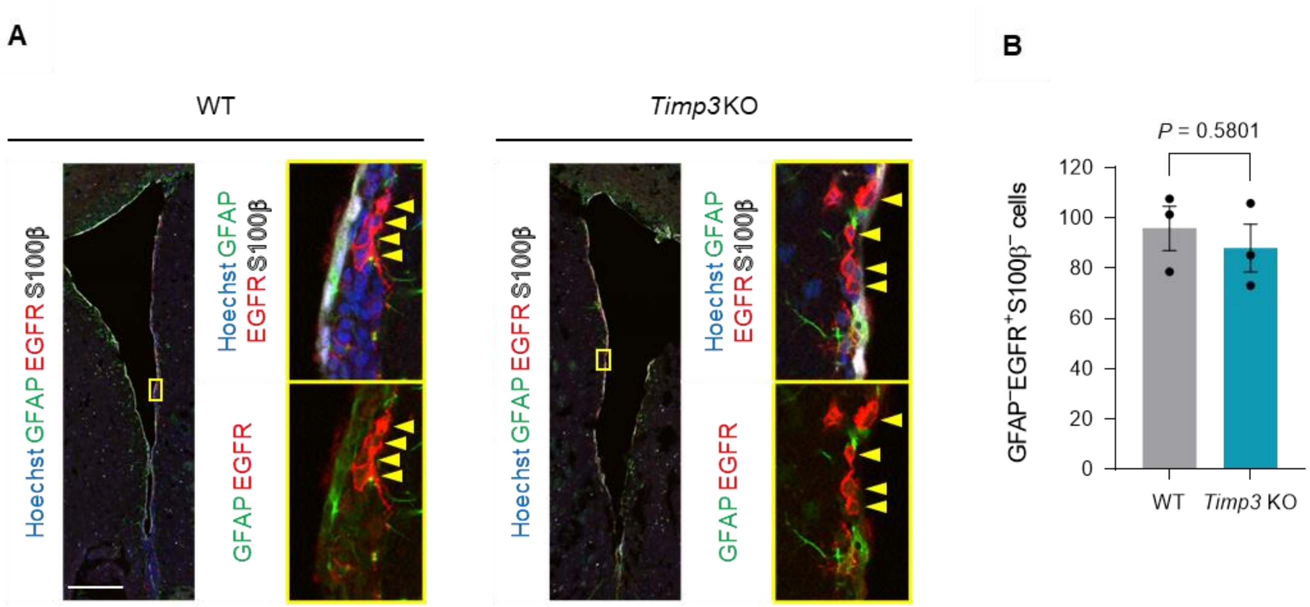

Supplementary Figure 1. Related to figure 3. (A) Immunohistofluorescence analysis of GFAP, EGFR, and S100β. Nuclei were stained with Hoechst 33342. Scale bar: 200 μm. Arrowheads indicate $GFAP^{-}EGFR^{+}S100\beta^{-}$ TAPs. (B) Quantification of $GFAP^{-}EGFR^{+}S100\beta^{-}$ TAPs. Data are means ± SEM (*n* = 3 and 3 animals for WT and *Timp3* KO, respectively). Two-tailed Student's *t* test.

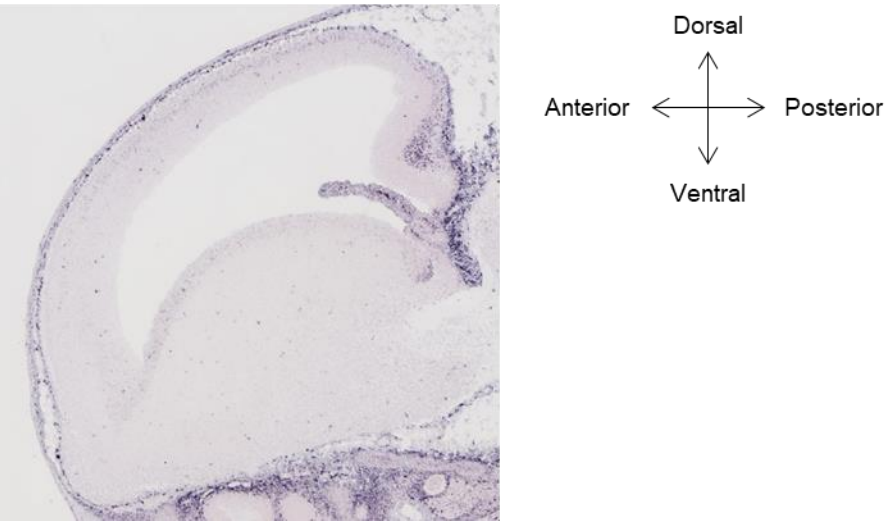

Supplementary Figure 2. Expression pattern of *Timp3* mRNA in E14.5 mouse forebrain. The image of *in situ* hybridization for *Timp3* was obtained from GenePaint (https://gp3.mpg.de/viewer/setInfo/EH1096/9).

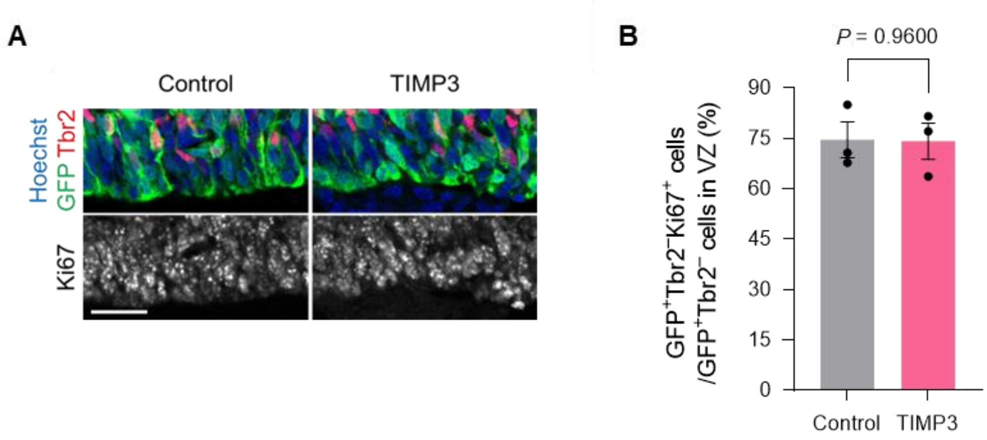

Supplementary Figure 3. Related to figure 6. (A) Embryos were subjected to immunohistofluorescence analysis of GFP, Tbr2, and Ki67 at E17.5. Nuclei were stained with Hochst 33342. Scale bar: 25 µm. (B) Quantification of the proportion of $Ki67^{+}$ cells among $GFP^{+}Tbr2^{-}$ cells in the VZ. Two-tailed Student's *t* test.

Gene ontology (biological process)	FDR
Dendrite development	3.4×10^{-8}
Regulation of cell morphogenesis	5.7×10^{-8}
Protein polyubiquitination	2.8×10^{-7}
Positive regulation of neuron differentiation	5.6×10^{-7}
Positive regulation of cell projection organization	5.6×10^{-7}
Axon development	1.1×10^{-6}
Synapse organization	1.1×10^{-6}
Regulation of mRNA metabolic process	5.4×10^{-6}
Proteasomal protein catabolic process	5.7×10^{-6}
Forebrain development	6.6×10^{-6}
Covalent chromatin modification	1.1×10^{-5}
Regulation of mitotic cell cycle	2.2×10^{-5}
Hippo signaling	2.2×10^{-5}
Small GTPase mediated signal transduction	2.8×10^{-5}
Regulation of cellular protein localization	3.2×10^{-5}
Neuron projection organization	5.5×10^{-5}
Epithelial tube morphogenesis	7.6×10^{-5}
Cell junction organization	1.8×10^{-4}
Protein modification by small protein removal	2.6×10^{-4}
Muscle tissue development	3.5×10^{-4}

Pathway	FDR
MicroRNAs in cancer	3.9×10^{-9}
FoxO signaling pathway	3.9×10^{-9}
Axon guidance	9.6×10^{-8}
Proteoglycans in cancer	5.5×10^{-7}
Adherens junction	3.3×10^{-6}
Prostate cancer	3.4×10^{-6}
Renal cell carcinoma	3.9×10^{-6}
mTOR signaling pathway	4.1×10^{-6}
MAPK signaling pathway	4.7×10^{-6}
Regulation of actin cytoskeleton	4.7×10^{-6}
Neurotrophin signaling pathway	4.7×10^{-6}
Pathways in cancer	1.0×10^{-5}
Ubiquitin mediated proteolysis	2.7×10^{-5}
Insulin signaling pathway	3.0×10^{-5}
EGFR tyrosine kinase inhibitor resistance	3.7×10^{-5}
Hippo signaling pathway	7.1×10^{-5}
Rap1 signaling pathway	7.4×10^{-5}
Protein processing in endoplasmic reticulum	7.7×10^{-5}
Hepatitis B	1.1×10^{-4}
Thyroid hormone signaling pathway	1.1×10^{-4}

Supplementary Figure 4. Gene ontology (biological process) and pathway (KEGG PATHWAY) enrichment analysis of TIMP3-expressing NPCs enriched genes (*edgeR*; $P < 0.05$). The top 20 are ranked by FDR.

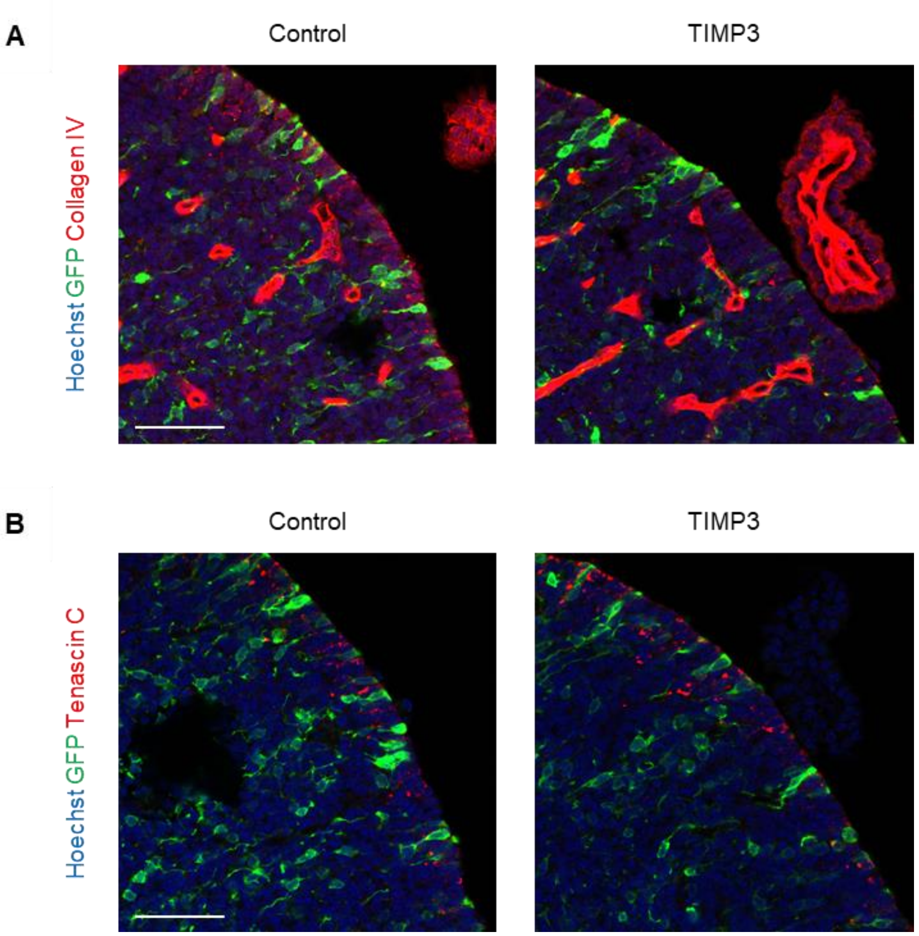

Supplementary Figure 5. *In utero* electroporation was performed at E13.5 with plasmids expressing GFP, alone (control) or together with TIMP3. Embryos were subjected to immunohistochemistry for GFP and Collagen IV (A) or Tenascin C (B) at E16.5. Nuclei were stained with Hoechst (blue).

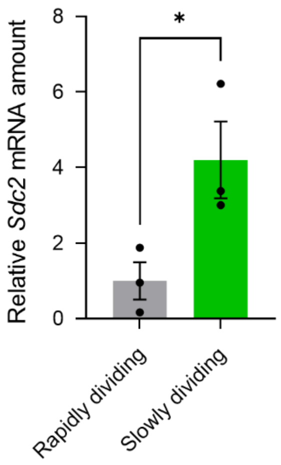

Supplementary Figure 6. *Sdc2* is highly expressed in slowly dividing NPCs. Quantitative RT-PCR analysis of *Sdc2* mRNA in rapidly and slowly dividing NPCs as defined in Figure 1. Data were normalized by the amount of *Actb* mRNA, and are means ± SEM (n = 3 independent experiments). *$P < 0.05$ by two-tailed Student's t test.

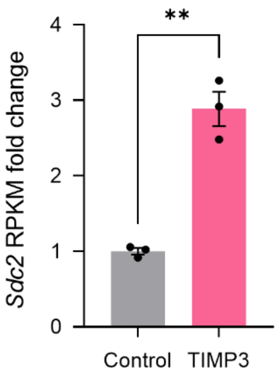

Supplementary Figure 7. TIMP3 overexpression upregulated *Sdc2* expression. *Sdc2* RPKM fold change relative to the average of control samples. Data are means ± SEM (n = 3 independent experiments). **P < 0.01 by two-tailed Student's t test.

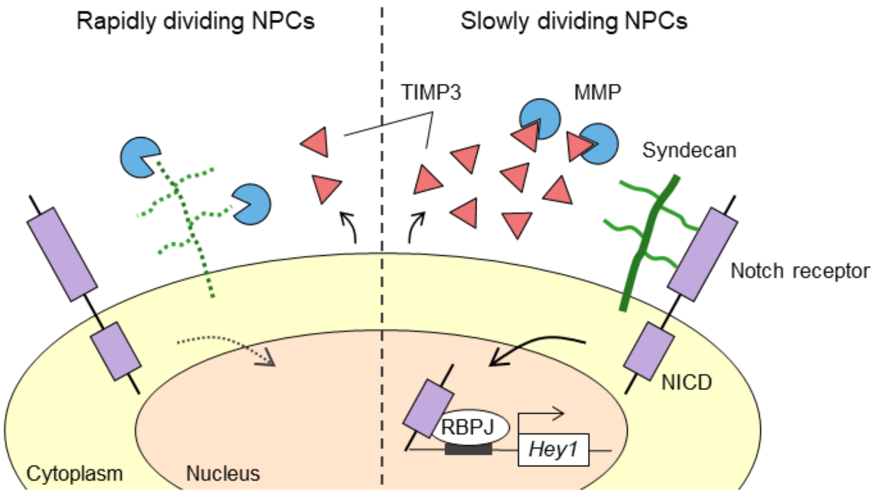

Supplementary Figure 8. Schematic model for the possible mechanism by which TIMP3 activates Notch signaling.

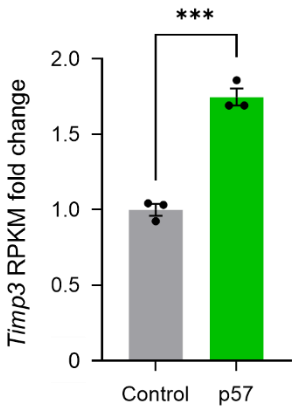

Supplementary Figure 9. p57 overexpression upregulated the expression level of *Timp3*. RNA sequencing data (Harada *et al*., 2021) were used to analyze the expression level of *Timp3*. Data are means ± SEM ($n = 3$ independent experiments). ***$P < 0.001$ by two-tailed Student's t test.
